# Acute Severe Autoimmune Hepatitis Presenting as Acute-on-Chronic Liver Failure Requiring Urgent Liver Transplantation: A Case Report

**DOI:** 10.7759/cureus.115485

**Published:** 2026-08-31

**Authors:** Tânia Pereira da Silva, Ana Correia De Sá, Ana Luis Ferreira, Sara Freitas, Glória Alves

**Affiliations:** 1 Internal Medicine, Unidade Local de Saúde do Alto Minho, Viana do Castelo, PRT; 2 Internal Medicine, Unidade Local de Saude do Alto Ave, Guimarães, PRT

**Keywords:** acute liver failure, acute-on-chronic liver failure, autoimmune hepatitis, liver transplantation, meld-na score

## Abstract

Autoimmune hepatitis (AIH) is classically a chronic immune-mediated inflammatory liver disease; however, it may present abruptly as acute severe hepatitis and, in a minority of cases, progress to liver failure. Early recognition and prognostic stratification are essential in severe presentations, as some patients may require urgent referral for liver transplantation. We report a 66-year-old female presenting with jaundice and marked hepatocellular injury who rapidly progressed to acute-on-chronic liver failure (ACLF) with multiorgan dysfunction. Despite supportive care and an extensive etiological workup, her condition deteriorated with hepatic encephalopathy, acute kidney injury consistent with hepatorenal syndrome, and infectious complications. A transjugular liver biopsy demonstrated intense inflammatory activity with prominent lymphoplasmacytic infiltrates and interface hepatitis, supporting AIH on a background of previously unrecognized chronic liver disease. Given poor prognostic indicators (Model for End-Stage Liver Disease-Sodium (MELD-Na) score >30 and ACLF grade 3), she was urgently referred to a transplant center and successfully underwent emergency liver transplantation. This case highlights the importance of maintaining a high index of suspicion for AIH in cases of acute liver injury of unclear etiology, performing early and serial prognostic reassessment, and expediting transplant evaluation when advanced organ failure develops.

## Introduction

Autoimmune hepatitis (AIH) is an immune-mediated necroinflammatory liver disease characterized by a combination of clinical, biochemical, serological, and histological features. Although AIH commonly follows a chronic course, its clinical spectrum is broad and includes acute hepatitis and, in rare cases, acute liver failure [1-3].

Acute severe autoimmune hepatitis (AS-AIH) represents a diagnostic and therapeutic challenge, as conventional autoantibodies may be absent or present at low titers in acute presentations, while liver histology may play a crucial role in establishing the diagnosis [1,3,4].

Acute-on-chronic liver failure (ACLF) is a distinct clinical syndrome characterized by acute decompensation in patients with chronic liver disease, accompanied by organ failure and high short-term mortality. Early recognition, appropriate risk stratification, and timely consideration of liver transplantation are essential in patients with progressive organ dysfunction [5,6].

In AS-AIH, corticosteroid therapy may lead to recovery in selected patients; however, severe coagulopathy, hepatic encephalopathy, elevated Model for End-Stage Liver Disease-Sodium (MELD-Na) scores, and multiorgan dysfunction are associated with poor response and should prompt early transplant evaluation [1,4,7,8].

We describe a case of previously undiagnosed AS-AIH presenting with ACLF and rapidly progressing to ACLF grade 3 with multiorgan dysfunction and bloodstream infection. The case is particularly challenging because of the very narrow therapeutic window for corticosteroid therapy, the rapid clinical deterioration, and the subsequent need for emergency liver transplantation.

## Case presentation

A 66-year-old female with a history of bilateral breast cancer presented to the emergency department with a two-week history of progressive asthenia, upper abdominal pain, and jaundice. She had been diagnosed with left breast cancer in France in 2006 and underwent breast-conserving surgery (lumpectomy), followed by chemotherapy and radiotherapy completed in 2006-2007. In 2021, she was diagnosed with right breast cancer, also in France, and underwent segmentectomy with negative surgical margins, followed by adjuvant radiotherapy. Since returning to Portugal, she has been under oncological surveillance at a private hospital, with no evidence of disease recurrence (last documented mammography in October 2023; Breast Imaging-Reporting and Data System (BI-RADS) 2 [9]). She denied recent travel, new prescribed medications, over-the-counter drugs, herbal supplements, or significant alcohol intake.

Initial laboratory evaluation at presentation revealed severe hepatocellular injury, with aminotransferases exceeding 20 times the upper limit of normal, conjugated hyperbilirubinemia, prolonged international normalized ratio (INR), and thrombocytopenia. Abdominal computed tomography demonstrated a dysmorphic liver with irregular contours and moderate ascites, raising suspicion of underlying chronic liver disease (Figure 1). She was admitted to an intermediate care unit for close monitoring and etiological investigation.

**Figure 1:**
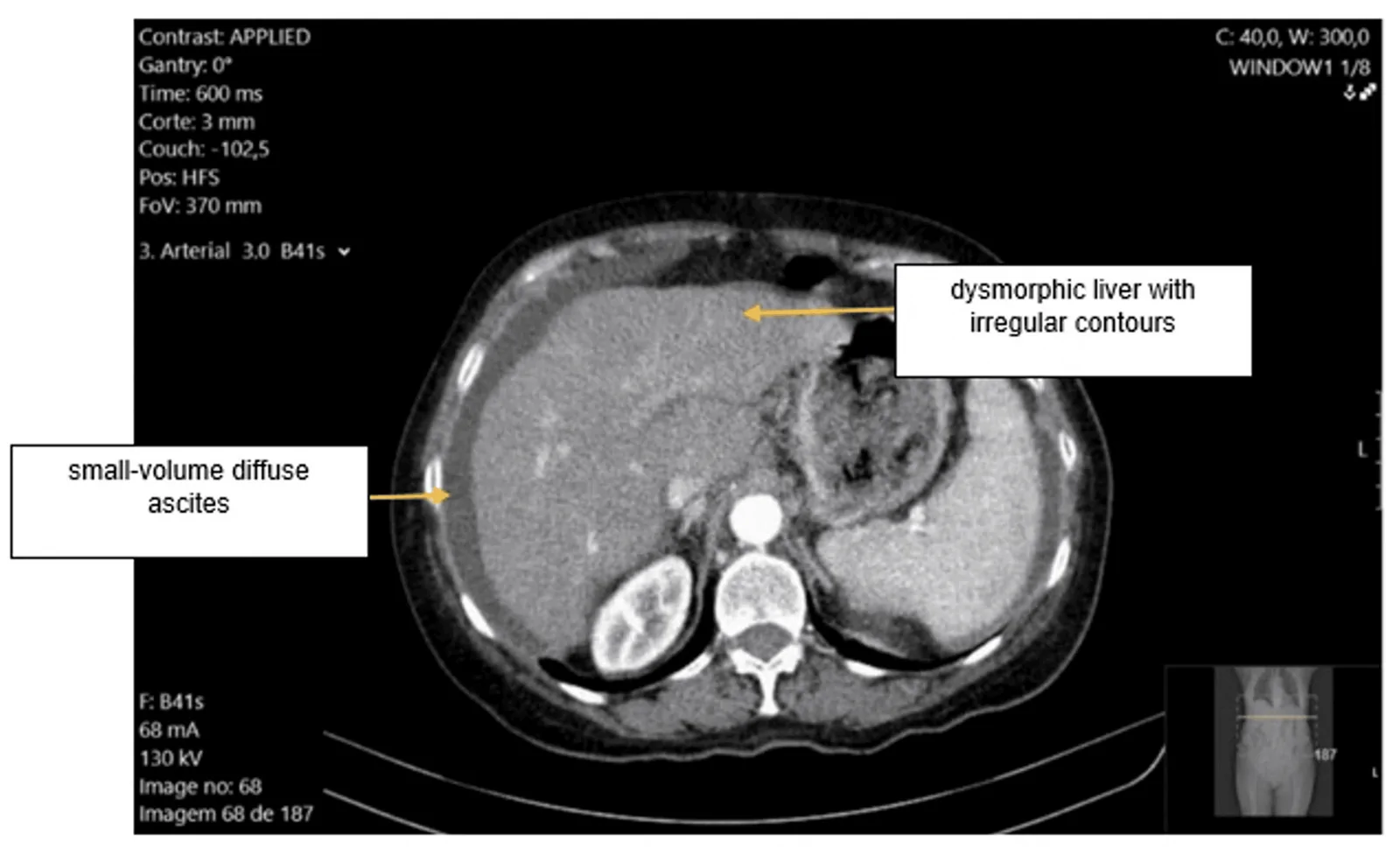
Abdominal computed tomography showing a dysmorphic liver with irregular contours, suggestive of chronic inflammatory hepatopathy, and small-volume diffuse ascites.

During hospitalization, the patient’s clinical condition deteriorated rapidly. She developed hepatic encephalopathy, worsening ascites, and oliguric acute kidney injury. By day 6 of hospitalization, her disease severity was reflected by a MELD-Na score of 34 [8] and a Chronic Liver Failure Consortium Acute-on-Chronic Liver Failure (CLIF-C ACLF) score of 56 [10]. At that time, laboratory findings included a total bilirubin of 20.78 mg/dL, INR of 2.5, creatinine of 1.47 mg/dL, and serum sodium of 131 mmol/L. Total bilirubin subsequently reached a maximum of 30.46 mg/dL, with a maximum INR of 2.7.

Although corticosteroid therapy would ordinarily be considered in AS-AIH, the therapeutic window for initiating immunosuppression in this patient was very short. The patient deteriorated rapidly, progressing to ACLF grade 3 with multiorgan dysfunction before corticosteroid therapy could be instituted. Consequently, there was no adequate opportunity for a corticosteroid trial before the clinical situation became dominated by advanced organ dysfunction and the need for urgent transplantation.

On day 7 of hospitalization, blood cultures became positive for *Staphylococcus epidermidis*, with detection of the mecA gene. This was interpreted as a possible bloodstream infection, and intravenous vancomycin was initiated. The patient continued antimicrobial treatment following transfer to the liver transplant center. The clinical progression and key management decisions are summarized in Table 1.

**Table 1:** Timeline of clinical progression and management decision. Abbreviations: MELD-Na, Model for End-Stage Liver Disease-Sodium; CLIF-C ACLF, Chronic Liver Failure Consortium Acute-on-Chronic Liver Failure; AIH, autoimmune hepatitis; IAIHG, International Autoimmune Hepatitis Group; ACLF, acute-on-chronic liver failure.

Timepoint	Clinical course/key findings	Management/decision
Pre-admission	Two-week history of asthenia, upper abdominal pain, and jaundice	—
Admission	Acute severe liver injury with jaundice, coagulopathy, and ascites; imaging suggestive of previously unrecognized chronic liver disease	Hospitalization and diagnostic workup
Days 1-3	Progressive hepatic dysfunction; autoimmune and infectious workup initiated	Supportive care and close monitoring
Day 4	Transjugular liver biopsy performed because of persistent diagnostic uncertainty and progressive liver dysfunction	Histopathological evaluation
Day 6	Hepatic encephalopathy, oliguric acute kidney injury, and worsening hepatic dysfunction; MELD-Na = 34; CLIF-C ACLF = 56	Intensive supportive management and ongoing discussion with transplant center
Day 7	Blood cultures positive for *Staphylococcus epidermidis*, with mecA detected; possible bloodstream infection	Intravenous vancomycin initiated
Day 10	Definitive liver biopsy result supported the diagnosis of AIH; simplified IAIHG score 6	Transferred to the liver transplant center
Days 10-12	Further clinical deterioration with advanced ACLF and multiorgan dysfunction	Intensive organ support and transplant evaluation
Day 12	Persistent ACLF grade 3 and poor short-term prognosis	Listed for urgent liver transplantation
Day 15	Persistent multiorgan dysfunction	Emergency orthotopic liver transplantation performed
Follow-up	Clinical recovery and progressive normalization of liver function	Post-transplant immunosuppressive therapy and follow-up

A comprehensive etiological workup excluded viral hepatitis (A, B, C, and E), cytomegalovirus, Epstein-Barr virus, metabolic liver disease, and drug-induced liver injury. Given persistent diagnostic uncertainty and progressive liver dysfunction with coagulopathy, a transjugular liver biopsy was performed on day 4 of hospitalization. The definitive histopathological result became available on day 10 and showed extensive multilobular and bridging confluent necrosis with collapse of the hepatic parenchyma, associated with dense lymphoplasmacytic inflammation, interface hepatitis, and prominent plasma cell infiltration (Figure 2). These findings, together with elevated IgG, positive anti-smooth muscle antibody (ASMA), and exclusion of viral hepatitis, supported the diagnosis of autoimmune hepatitis. The simplified International Autoimmune Hepatitis Group (IAIHG) score was 6 points [11].

**Figure 2:**
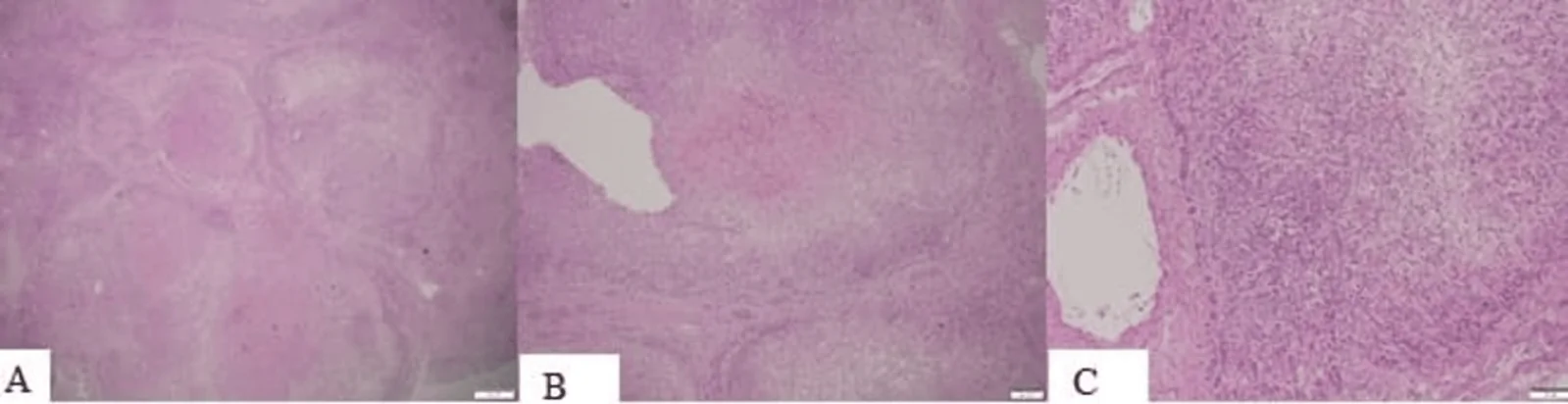
Histopathological findings from the transjugular liver biopsy (hematoxylin and eosin staining). (A) Low-power view (2× magnification) showing extensive multilobular/bridging confluent necrosis with collapse of the hepatic parenchyma. (B) Intermediate-power view (4× magnification) demonstrating hepatocellular necrosis associated with dense lymphoplasmacytic inflammatory infiltrate. (C) High-power view (10× magnification) highlighting interface hepatitis with prominent plasma cell-rich inflammatory infiltrates and ongoing hepatocyte injury, supporting the diagnosis of severe autoimmune hepatitis presenting as acute-on-chronic liver failure. Images kindly provided by Dr. José Ramón Vizcaíno, Department of Pathology, Unidade Local de Saúde de Santo António, Porto, Portugal.

The diagnostic workup and key findings supporting the final diagnosis are summarized in Table 2.

**Table 2:** Diagnostic workup for acute liver injury and evaluation for autoimmune hepatitis. The Simplified International Autoimmune Hepatitis Group (IAIHG) score was calculated according to the published criteria [11]. Abbreviations: CMV, cytomegalovirus; EBV, Epstein–Barr virus; HIV, human immunodeficiency virus; IgG, immunoglobulin G; ASMA, anti-smooth muscle antibody; C4, complement component 4; ANA, antinuclear antibody; anti-LKM-1, anti-liver/kidney microsomal antibody type 1; anti-SLA/LP, anti-soluble liver antigen/liver-pancreas antibody; anti-LC-1, anti-liver cytosol type 1 antibody; AMA-M2, antimitochondrial antibody M2; AST, aspartate aminotransferase; ALT, alanine aminotransferase; ALP, alkaline phosphatase; GGT, gamma-glutamyl transferase; LDH, lactate dehydrogenase; INR, international normalized ratio; CT, computed tomography; IAIHG, International Autoimmune Hepatitis Group.

Investigation	Key findings	Interpretation
Viral hepatitis (A, B, C, and E)	Serological testing negative	Viral etiology excluded
CMV, EBV, and HIV	No evidence of active infection	Infectious etiology excluded
Metabolic workup (iron and copper studies)	No diagnostic abnormalities	Metabolic liver disease excluded
Drug/toxin exposure	No relevant exposure identified	Drug-induced liver injury unlikely
Autoimmune markers	IgG: 2889 mg/dL (reference: 700-1600 mg/dL); ASMA: positive, titer 1:40; C4: decreased (reference: 10-40 mg/dL); ANA, anti-LKM-1, anti-SLA/LP, anti-LC-1, and AMA-M2 negative (reference: negative)	Findings supportive of autoimmune hepatitis
Liver biochemistry at transfer to the liver transplant unit	AST: 298 U/L (reference: 10-40 U/L); ALT: 188 U/L (reference: 7-56 U/L); total bilirubin: 30.46 mg/dL (reference: 0.1-1.2 mg/dL); direct bilirubin: 19.43 mg/dL (reference: <0.3 mg/dL); ALP: 99 U/L (reference: 44-147 U/L); GGT: 166 U/L (reference: 9-48 U/L); LDH: 331 U/L (reference: 140-280 U/L)	Acute hepatocellular injury pattern with marked hyperbilirubinemia
Coagulation	INR: maximum 2.7	Severe hepatic dysfunction
Imaging (CT scan)	Dysmorphic liver and ascites	Findings suggestive of underlying chronic liver disease
Liver biopsy	Interface hepatitis with plasma cell infiltration	Findings consistent with autoimmune hepatitis
Simplified IAIHG score	Immunoglobulin G (IgG) >1.1× upper limit of normal (2 points), anti-smooth muscle antibody (ASMA) titer 1:40 (1 point), liver histology compatible with autoimmune hepatitis (1 point), and absence of viral hepatitis (2 points), resulting in a total score of 6 points	Findings fulfilling diagnostic criteria for autoimmune hepatitis

The patient progressed to ACLF grade 3 with multiorgan dysfunction, including hepatic encephalopathy, oliguric acute kidney injury consistent with hepatorenal syndrome, severe hepatic dysfunction, and coagulopathy. On day 6, her MELD-Na score was 34, and her CLIF-C ACLF score was 56, indicating a very high short-term mortality risk. She subsequently developed a possible bloodstream infection on day 7, further limiting the potential benefit and safety of immunosuppressive therapy.

Although corticosteroid therapy would ordinarily be considered in AS-AIH, the rapid clinical deterioration resulted in a very narrow therapeutic window, and corticosteroid therapy could not be initiated before the patient progressed to advanced ACLF with multiorgan dysfunction. The subsequent bloodstream infection further reinforced the decision to avoid immunosuppression and prioritize definitive transplantation.

The transplant team was involved from an early stage because of the progressive clinical deterioration. Following the availability of the definitive liver biopsy result on day 10, which supported the diagnosis of autoimmune hepatitis, the patient was transferred to the liver transplant center for further evaluation. She was formally listed for urgent liver transplantation on day 12 because of persistent ACLF grade 3, multiorgan dysfunction, and poor short-term prognosis. She underwent emergency orthotopic liver transplantation on day 15 for chronic liver disease of autoimmune etiology with hepatic failure and ACLF grade 3. Following transplantation, she had a favorable postoperative course with progressive clinical recovery.

## Discussion

This report illustrates a rare but clinically critical presentation of AIH as acute severe hepatitis evolving into ACLF with multiorgan dysfunction. Current guidance underscores that AIH may present across a broad clinical spectrum, including severe acute hepatitis and acute liver failure, and that diagnostic certainty may be challenging early in the disease course [1-3]. In acute presentations, conventional serological markers may be absent or atypical, and liver histology can play a central role in supporting the diagnosis and excluding alternative etiologies [1,3-5].

ACLF represents a high-risk phenotype of acute decompensation in patients with underlying chronic liver disease, characterized by organ failure and high short-term mortality. European guidelines emphasize prompt recognition, early identification and management of precipitating factors, including infection, appropriate organ support, and expedited assessment for liver transplantation in eligible patients [12-14]. In our patient, the combination of hepatic encephalopathy, renal dysfunction with oliguric acute kidney injury, coagulopathy, and subsequent possible bloodstream infection reflected severe systemic illness and advanced ACLF. The presence of a dysmorphic liver, irregular hepatic contours, and ascites at presentation further supported the presence of previously unrecognized chronic liver disease.

The role of corticosteroids in AS-AIH remains important but must be individualized according to disease severity, treatment response, and the presence of contraindications or competing risks. Current guidance supports corticosteroid therapy in appropriately selected patients with AS-AIH, with close early reassessment and prompt transition to a transplant pathway when there is inadequate clinical or biochemical response [1,2]. Observational studies and multicenter data indicate that greater baseline disease severity, including higher MELD-based scores, hepatic encephalopathy, and ascites, together with failure to demonstrate early improvement, is associated with poor corticosteroid response and adverse outcomes [4,5,7].

In our patient, corticosteroid therapy was not initiated because the therapeutic window for a trial of immunosuppression was very short. The patient deteriorated rapidly from acute severe hepatitis to ACLF grade 3 with multiorgan dysfunction before corticosteroid therapy could be instituted. By day 6 of hospitalization, the MELD-Na score was 34, and the CLIF-C ACLF score was 56, reflecting severe disease and a very poor short-term prognosis. The subsequent development of possible bloodstream infection on day 7, with blood cultures positive for *Staphylococcus epidermidis* and detection of the mecA gene, further increased the risks associated with immunosuppression. Intravenous vancomycin was initiated and continued following transfer to the liver transplant center. In this setting, there was no adequate opportunity for a corticosteroid trial, and the clinical priority shifted toward definitive transplantation rather than delaying transplantation to attempt immunosuppressive therapy. The patient was transferred to the liver transplant center on day 10, listed for urgent transplantation on day 12, and underwent emergency liver transplantation on day 15 [5,7,12-14].

The severity of the clinical course was further reflected by ACLF grade 3, with hepatic encephalopathy, oliguric acute kidney injury consistent with hepatorenal syndrome, severe hepatic dysfunction, and coagulopathy. Total bilirubin subsequently reached 30.46 mg/dL, and INR was 2.7. Although the patient required only low-flow oxygen supplementation (2 L/min) before transfer, her condition deteriorated further after transfer, ultimately requiring continuous renal replacement therapy and mechanical ventilation. These features highlight the rapidly progressive nature of the disease and the narrow window available for therapeutic intervention.

Overall, this case reinforces three practical messages: (1) AIH should be considered early in the differential diagnosis of acute liver injury of unclear etiology, including in older adults without previously recognized chronic liver disease; (2) prognostic assessment should be performed early and repeated throughout the clinical course to guide treatment escalation; and (3) when progressive organ failure develops, particularly hepatic encephalopathy and renal dysfunction, timely transplant referral should proceed in parallel with supportive care, management of precipitating factors, and etiological clarification [1-3,5-7,12-14].

In selected patients with advanced ACLF and high MELD scores, early liver transplantation may provide a substantial survival benefit compared with continued medical management [12,13]. In this case, the MELD-Na score of 34, CLIF-C ACLF score of 56, ACLF grade 3, rapid clinical deterioration, and multiorgan dysfunction supported the need for urgent transplantation. The patient underwent emergency liver transplantation with a favorable outcome.

## Conclusions

AS-AIH may present abruptly and rapidly progress to ACLF with multiorgan dysfunction. Early recognition, often supported by liver biopsy, together with serial prognostic assessment, is crucial. In patients with advanced organ dysfunction and poor prognostic indicators, timely referral to a transplant center is essential, as liver transplantation may represent the only life-saving therapeutic option.
